# Use of Pilocarpinum Muriaticum in Children’s Diseases

**Published:** 1879-08

**Authors:** 


USE OF PILOCARPINUM MURIATICUM IN CHILDREN'S DISEASES.—Weiss (*Pest. Med. Chir. Presse*, 1879—2) has had the opportunity of observing the effects of pilocarpine in fourteen cases where the patients were suffering from nephritis, complicated with general dropsy, following scarlatina. In four cases there existed extensive bronchitis, in two diphtheria, and in one pneumonia of the left side of the lung. In each of these cases the results produced by pilocarpine were most favorable, and the patients could all be dismissed as cured. One of the most important properties of pilocarpine is that it prevents the dropsy from increasing, keeping it stationary without implicating the kidneys, till the latter have recovered their power of secreting urine more abundantly. Two different kinds of solution where used for the hypodermic injections; a 1 per cent. solution for children under four years, and a 2 per cent. one for children above four years. In such young patients, where collapse seemed to threaten from prolonged illness and great weakness, four or five drops of ether were added to the solution of pilocarpine in the syringe. The author observed that whenever he used this mixture, the young patients did not present the phenomena which generally followed the injection of a solution of pure pilocarpine; viz., vomiting, nausea, hiccough, pallor, and a feeble pulse. The injections were made once daily into the upper arm, beginning with half a syringeful, and rising to a whole one. The effects of pilocarpine generally appeared after a few minutes, beginning with a slight flush on the face, which, however, gradually increased, and only disappeared when the perspiration had ceased. The latter set in after three to five minutes, beginning on the forehead and face, and gradually spreading over the rest of the body. The duration of the perspiration was different; in one case it lasted for one-and-a-half hours, in another, three-and-a-half hours, in a third case, of very considerable universal dropsy, where the amount of urine passed in the twenty-four hours was only one hundred and fifty ccm., the secretion lasted for fifteen hours, after which, the œdematous infiltration decreased considerably. The quantity of fluid secreted in the saliva and the perspiration were in direct proportion to the amount of pilocarpine which had been injected, and to the strength of the solution. Thus, a two per cent. solution always called forth a more considerable secretion of perspiration and saliva than a one per cent. solution. Two out of the fourteen patients complained in the abdomen after the injection, and four of headache. In eight cases, the pupil was seen to contract; the contraction began at the same time at which perspiration set in, and lasted from thirty to forty-five minutes. The temperature was taken in every case both before and after the injection, and in several of them was observed to fall rapidly after the injection; this decrease, however, never lasted longer than from half an hour to three hours, after which time the one case, where the perspiration had lasted for sixteen hours, the temperature, which had been 40.4 deg. Cent. before the injection, fell to 38.6 thirty-five seconds after it, and did not rise again. The pulsations of the radial artery increased in a minute from 12 to 30; the pulse was full and jerking; this acceleration lasting from fifteen to thirty minutes, after which time the pulse regained its previous character. In four cases, the patients vomited. The vomited matter consisted mostly of mucus. After the injection, almost all the children coughed very much; in four cases where there was extensive bronchitis, and in a fifth, which had been showing symptoms of œdema of the lungs and uræmia, the lungs were entirely cleared from the secretion which had accumulated in them by the frequent coughing within forty-eight hours. In nine cases, there was a strong desire to micturate immediately after the injection; and, in three, to evacuate the bowels. The motions were thin and very offensive, and were passed in great quantity. In a case of constipation which had lasted four days, the bowels were moved copiously immediately after the injection.

There were no notable increase in the quantity of urine passed after pilocarpin had been injected; it was of a much lighter color than before. The following are the author's conclusions: 1. Pilocarpine has proved to be a very successful remedy for children who suffer from nephitis and scarlatina; 2. In giving it to children, care should be taken to begin at first with small doses, which may later on be gradually increased; 3. If the little patients are very weak and are likely to collapse after the injection, a few drops of ether should be added to the pilocarpine solution ; 4. The drug produces a very copious and lasting secretion of sweat, such as no other drug ever has been known to call forth—it acts quickly; 5. In cases of bronchitis, complicated by dropsy, which often produce dyspnœa in children, the affection of the bronchii vanishes very soon after the remedy has been administered.—*London Medical Review.*

